# Subtractive color filters based coaxial metasurface structures with high saturation and brightness

**DOI:** 10.1038/s41598-026-51341-0

**Published:** 2026-05-13

**Authors:** Abdelnaser Ali, Hassan Sayed, Mohamed Mobarak, Arafa H. Aly, Walied Sabra

**Affiliations:** 1 TH-PPM Group, Physics Department, Faculty of Science, Beni-Suef University, Beni Suef 62511, Egypt; 2 Physics Department, Faculty of Science, Beni-Suef University, Beni Suef 62511, Egypt

**Keywords:** Metasurface, Coaxial aperture, Surface plasmon resonance, Subtractive color filter, Engineering, Materials science, Optics and photonics, Physics

## Abstract

We propose and numerically demonstrate subtractive color filters (SCFs) based on a plasmonic metasurface including coaxial aperture geometry working in the visible range. The Proposed designs involve arranging coaxial apertures (circular, elliptical, square, rectangular) in a silver film for reflection mode color filtering. By controlling the geometric parameters of the SCFs design, we achieve tunability of high-absorption resonance peaks (> 99.9%) across the visible spectrum. The proposed SCFs are supposed to exhibit a polarization-insensitive operation for symmetric apertures and a polarization-sensitive response for asymmetric apertures. Furthermore, the devices provide a relaxed angular tolerance. Finally, our results are then modeled using nonlinear regression analysis to a cubic regression, and a quadratic exponential model. Accordingly, it is possible to create an SCF for any color of choice in a straightforward way. This technique constitutes a promising process that provides a methodology to design color filters for practical applications such as color printing, high-resolution chromatic displays, and multispectral imaging.

**Supplementary Information:**

The online version contains supplementary material available at 10.1038/s41598-026-51341-0.

## Introduction

Color filters have been widely applied in several applications, due to the importance in the optical devices. Such as, color display, photovoltaic devices, photography, optical sensing, hologram projection, and optical measurement devices^1–9^. In the past, the optical properties of the traditional dye-based color filter work based on the chemical bonds. These Organic color filters suffer from limited thermal and photochemical stability, and environmental hazards^10^. The organic polymers like polyphenylene sulfide (PPS) exhibit significant color changes under heat and light due to polyconjugated structure formation, resulting in limitations in high load optical applications such as color filters in projection displays^11^. For instance, even state-of-the-art photostable dyes like $\:{\mathrm{H}\mathrm{M}\mathrm{S}i\mathrm{R}}_{680}$-Me have been reported to lose approximately 47% − 51% of their signal intensity after only one hour of high-intensity^12^. The emerging structural color filters have drawn a lot of interest as nanotechnology has advanced^13–22^. The working principle of this type of color filter depends on the interaction between the incident electromagnetic waves and nanostructures. The colorant nanotechnology-based structures have many advantages, for example, they are able to overcome the drawbacks of conventional dye-based color filters and fade resistance^3,10^. Recently, plasmonic color filters have been developed and their performance enhanced using various types of structures and design methodologies. These various types of plasmonic color filters include a combination of metal/insulator/metal^23–25^, plasmonic gratings^26–29^, metallic Nanoholes arrays^30–32^, nanorod structures^33^, plasmonic color filters based on liquid crystal structures^34^, and nanovolcano arrays^35^. Beyond color filtering, metasurfaces have demonstrated extraordinary capabilities in various photonic applications due to their ability to manipulate electromagnetic waves at the subwavelength scale. For instance, suggests potential applications in signal sensing, solar thermal harvesting, electromagnetic wave absorption, refractive index sensing, and optical switching^36–38^. Recent works have demonstrated multichannel plasmonic color filters with additional functionalities such as optical sensing, switching, and high-resolution imaging^39,40^. Previous investigations have demonstrated that these plasmonic modes are highly sensitive to geometric configurations. For instance, silver-shell nanopearls and nanocylinder arrays exhibit tunable SPR responses such as bonding and anti-bonding modes and spectral redshifts that are absent in solid metallic structures^41,42^. Furthermore, 3D analyses of silver nanoparticle systems emphasize the critical role of polarization and particle size in modulating optical field distributions^43^. These fundamental insights into plasmonic tunability and field confinement provide the physical justification for the silver-based coaxial aperture structures proposed in this work. Among these structures, annular aperture arrays (AAAs)^44^, that confine light into nanometric volumes, giving demonstrated unique capabilities in controlling optical transmission and reflection, enabling applications in plasmonics, biochemical sensing, imaging, optical trapping, nonlinear optics, nanophotonics, and color filtering^45–53^. Depending on the plasmonic structure, either localized surface plasmons or surface plasmon polaritons create resonant absorption as a result of the interaction between light and surface plasmons in these structures. In plasmonic devices, this resonant absorption may be utilized to create subtractive color filters (SCFs). SCFs for colors such as cyan, magenta, and yellow can achieve higher absorption/reflection efficiency since they work based on the removal of specific wavelengths within the visible spectrum, depending on the design parameters, while the remaining spectrum combined to achieve these colors^54–57^. Traditionally, most research on (AAAs) has focused on the transmission mode, where light passes through nanostructured metallic films patterned with coaxial ring apertures. This mode has revealed resonance behaviors driven by localized surface plasmon resonances (LSPRs) and surface plasmon polaritons (SPPs). Particularly when using noble metals such as gold and silver^58,59^. While transmission-based studies have led to various developments in optical filtering and plasmonic color displays^60^, the reflection mode remains underexplored, especially for coaxial aperture geometries, as same as the transmission mode. Recently, phase-change materials have been classified as an alternative material for functional tunability in nanophotonic devices. For example, an atomic rearrangement metamaterial (ARM) enables continuous refractive index modulation through thermal excitation^61^. While Sb₂Se₃ and Sb₂S₃ thin films demonstrate that controlled doping and interface engineering can substantially alter optoelectronic performance^62,63^.

In our study, we propose and investigate an approach to achieve subtractive color filtering based on plasmonic metasurfaces by employing silver-based aperture arrays with various geometries (elliptical, circular, square, and rectangular) in the reflection mode. Then, the numerical simulations were performed by using the Finite Element Method in COMSOL Multiphysics software, Wave Optics Module, version 6.1^64^. As a result of removing the complementary components of blue, green, and red from the spectrum of the visible light, the proposed subtractive colors for yellow, magenta, and cyan are achieved, respectively, after combination of the remaining spectrum. The selected ranges for bluish, greenish, and reddish parts of the visible spectrum are (420–490 nm), (495–570 nm), and (580–660 nm), respectively^65,66^. This work presents the first systematic investigation of reflection-mode coaxial aperture metasurfaces as subtractive color filters across multiple geometries, complemented by analytical design equations that enable predictive color tuning without numerical optimization.

## Results and discussion

**Fig. 1:**
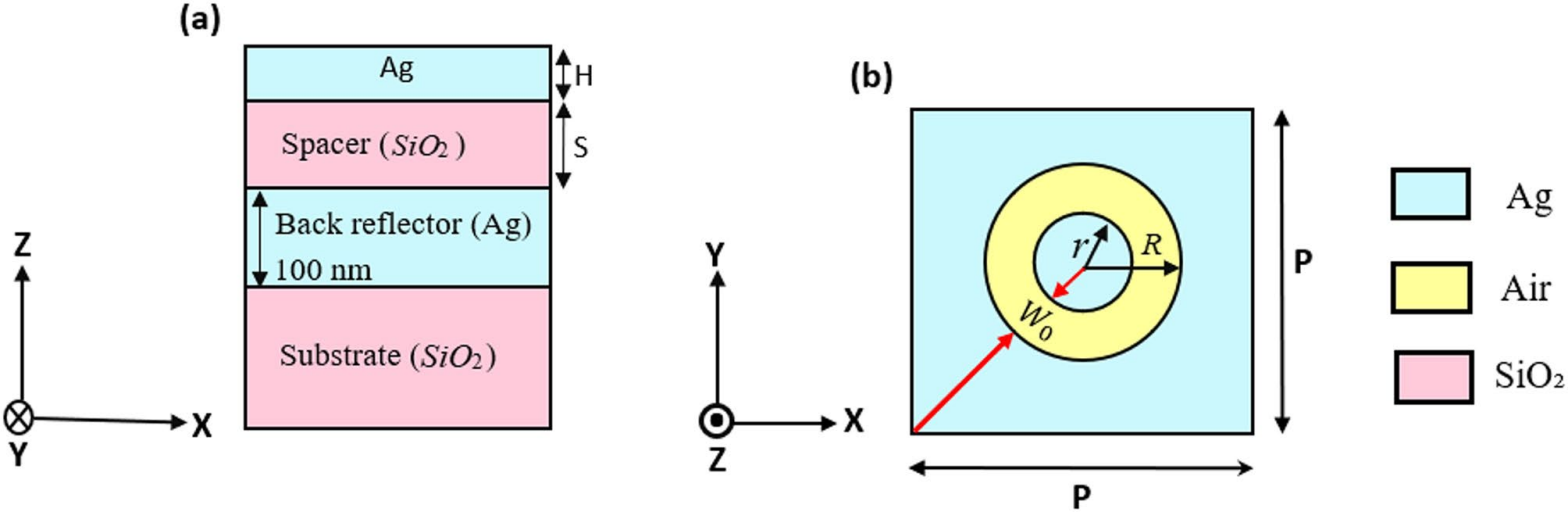
Schematic diagrams of (a) side and (b) top views of the unit cell of the proposed coaxial aperture structure for the proposed SCFs. The coaxial nano-apertures are formed by two concentric circles with outer and inner radii *R* and *r* (air cavity width $\:{w}_{0}$), respectively. H is the thickness of the Coaxial nano-apertures array (cavity depth), and S is the dielectric spacer thickness.

The initial structure of the suggested SCF design and the top view of the unit cell are shown in Fig. 1. The proposed SCF structure consists of an annular apertures array in silver (Ag) film, followed by a dielectric spacer layer made of SiO₂. A 100 nm-thick silver back reflector on a top of a SiO₂ substrate is introduced to ensure that the structure operates within the reflection mode. The electron beam lithography (EBL) process may be used to fabricate high optical quality sub-wavelength cylindrical and annular aperture arrays^67,68^. The spacer refractive index and the permittivity data for silver are obtained from Refs.^69,70^, respectively. These annular apertures can support two key surface plasmon modes. The first, cylindrical (cavity) surface plasmons (CSPs) circulate the inner sidewall of the ring, which affected by the geometrical design and the thickness of the metal film. The second one is the planar surface plasmons (PSPs), which propagate along the surface plane between the ring and the metal interface, which is mainly related to the periods of two-dimensional gratings fabricated therein^71–74^.

The structure is illuminated from the top by applying periodic boundary conditions along the x and y directions. Every SCF can be obtained at different parameters, including the periods of the square unit cells (P), spacer thickness (S), outer radius (R), inner radius (r), and the depth (H) of the silvered circular aperture array.

**Fig. 2:**
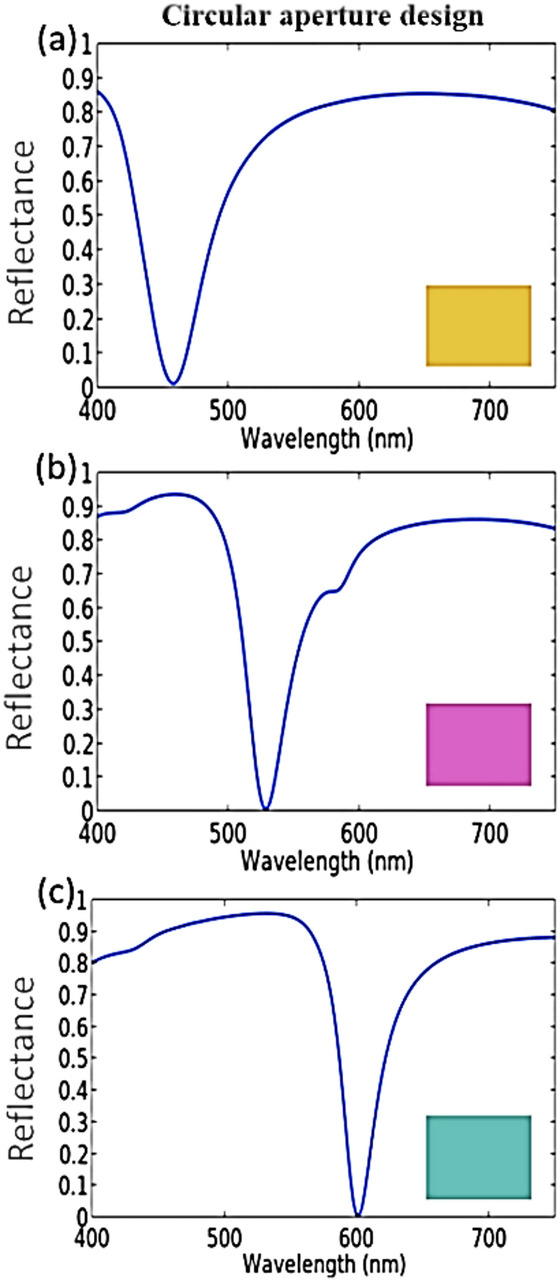
The obtained reflectance spectra for the suggested SCFs with circular aperture metasurfaces in Ag film that produce (**a**) yellow, (**b**) magenta, and (**c**) cyan colors.

In our process, the mentioned variables are considered as design parameters that can control the reflected spectra of the achieved SCFs. For yellow SCF, the structure parameters values are 175 nm, 95 nm, 25 nm, 65 nm, and 50 nm for P, S, H, R, and r, respectively. Also, the optimized parameters for magenta and cyan SCFs are (*P* = 300 nm, S = 125 nm, H = 25 nm, *R* = 80 nm, *r* = 55 nm) and (*P* = 300 nm, S = 153 nm, H = 25 nm, *R* = 70 nm, *r* = 55 nm), respectively, as shown in Fig. 2.

The yellow SCF in Fig. 2(a) arises from the combination of reflected green and red spectrums after subtracting the absorption peak resonance at 458 nm in the visible band. The magenta filter in Fig. 2(b) is produced by the combination of reflected blue and red wavelengths, where the absorption peak resonance is obtained at 529 nm. In Fig. 2(c), the combination of the reflected blue and green spectrums gives the cyan SCF with an absorption resonance peak at 602 nm. It is worth mentioning that the resulted SCFs exhibit strong absorption resonance peaks (≥ 99%), which in turn result in a very good saturation of subtractive colors. The absorption peak resonance can be obtained from the following formula: A = 1-R-T. The parameters A, R, and T are representing absorption, reflection, and transmission, respectively. To obtain absorption near unity, both the reflectance and transmittance need to be reduced to near zero. The transmittance T is zero since the thickness of the silver back reflector layer is 100 nm, which is greater than the skin depth of the silver film. As a result, A = 1-R. The reflected energy would only be zero under the condition of impedance matching (Z = 1), resulting in the perfect absorption of A ~ 100%. Where the impedance approaches that of free space, the electric displacement current density ( $\:{\mathrm{J}}_{\mathrm{d}}=$-iωεE, with ω is the operating radian frequency, ε is the local permittivity, and E is the local electric field) reaches its maximum as shown in Supplementary Fig. S1. The impedance Z of the metasurface structures can be obtained from the following equation^75^.

1 $$\:Z=\sqrt{\frac{1+{S}_{11}^{2}-{S}_{21}^{2}}{1-{S}_{11}^{2}-{S}_{21}^{2}}}$$

Here, $\:{S}_{11}$ and $\:{S}_{21}$ is the complex reflection and transmission coefficients, respectively.

To demonstrate the competitive performance of our proposed design, a quantitative comparison between our circular coaxial aperture and other previous literatures is summarized in Table 1. Our results show notably deeper resonance efficiency and a superior degree of color purity$\:{(P}_{e})$ and brightness, a relatively small bandwidth (FWHM), and sRGB Coverage.

**Table 1:** Performance comparison of the proposed circular coaxial aperture design with previously reported SCFs.

Ref.	Mode(T/*R*)		Absorption efficiency (%)	FWHM (nm)	Brightness	$\:{P}_{e}$ (%)	sRGB coverage (%)
Our work	R	Y	98.81	55.33	77.04	56.50	8.16
		M	99.41	41.12	54.51	34.69	
		C	99.66	33.90	73.95	14.52	
^18^	R		≥ 97	NG	NG	NG	NG
^54^	R		near-zero reflection dip at resonance	NG	NG	NG	NG
^55^	T		exceeds 90%.	NG	NG	NG	NG
^56^	R		up to 70%	⁓55	NG	NG	NG

NG = not given, T = transmission, and R = reflection. Y, M, C for yellow, magenta, and cyan colors, respectively.

**Fig. 3:**
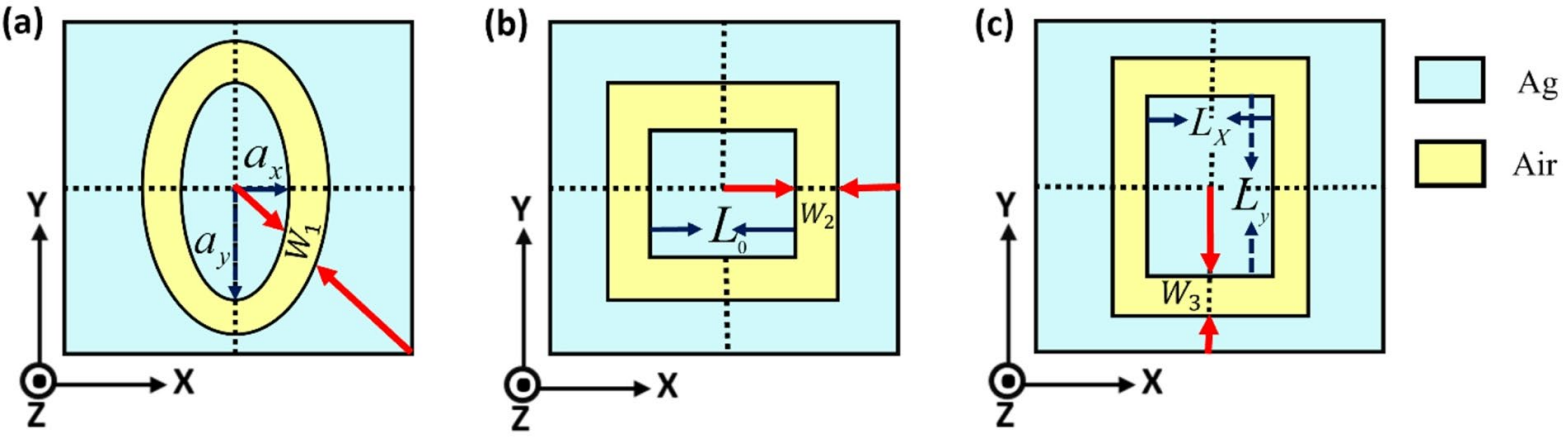
Concentric coaxial apertures of various geometries in Ag film with thickness (gap depth) H. (**a**) Elliptical aperture formed by two concentric ellipses with inner semi-axes $\:{a}_{x}$ and$\:{a}_{y}$, respectively (air gap width $\:{w}_{1}$). (**b**) Square aperture formed by two concentric squares with inner side length $\:{L}_{0}$ (air gap width $\:{w}_{2}$), and (c) Rectangular aperture formed by two concentric rectangles with inner width $\:{L}_{x}$ and length $\:{L}_{y}$ (air gap width $\:{w}_{3}$).

Additionally, we demonstrate the polarization-dependent response of the SCFs by using plasmonic nano-apertures of various forms in metasurface structures. In particular, we investigate the optical properties of elliptical, square, and rectangular aperture arrays embedded in a silver film, as illustrated in Fig. 3. For elliptical apertures, the inner semi-major and semi-minor axes in the x and y directions are denoting as $\:{a}_{y}$ and $\:{a}_{x}$, respectively with an air nano-cavity of width $\:{w}_{1}$​ separating the concentric inner and outer ellipses. For the square aperture, the inner side length is $\:{L}_{0}$ and the air gap width between the two concentric squares is $\:{w}_{2}$​. For the rectangular aperture, the inner width and length in the x and y directions are $\:{L}_{x}$ and $\:{L}_{y}$, the air gap width is $\:{w}_{3}$ sandwiched between the two concentric rectangles. In all cases, the nanoslit (or nanogap) depth is denoted by H, and the y-axis represents the direction of the E-field.

The geometric parameters directly influence the resonance behavior and color saturation of the filters. For circular apertures, increasing the outer radius $\:R$ ($\:{w}_{1}$ constant) extends the propagation distance for CSPs, shifting the resonance toward the longer wavelengths. A narrower gap enhances field confinement, sharpening the resonance dip and improving color saturation^76,77^. For elliptical apertures, the aspect ratio $\:({a}_{y}/{a}_{x})$ controls polarization splitting. The larger ratio creates a difference in the effective optical path along the two axes. This is resulting in color purity improvement along the major axis due to the extended path. For square apertures, the increase in the side length $\:{L}_{0}$ produces a red shift resonance. The sharp corners act as scattering centers that excite the localized surface plasmon resonances (LSPRs), improving the resonance and the resulting color purity. For rectangular apertures, a higher aspect ratio ($\:{L}_{y}/{L}_{x})$ increases the path anisotropy. This is supporting polarization splitting and providing high color purity for the long-axis polarization. However, it is dropping for the short axis making these apertures suitable for polarization-switchable devices. Additionally, the sharp corners further excite LSPRs, enhancing spectral selectivity.

**Fig. 4:**
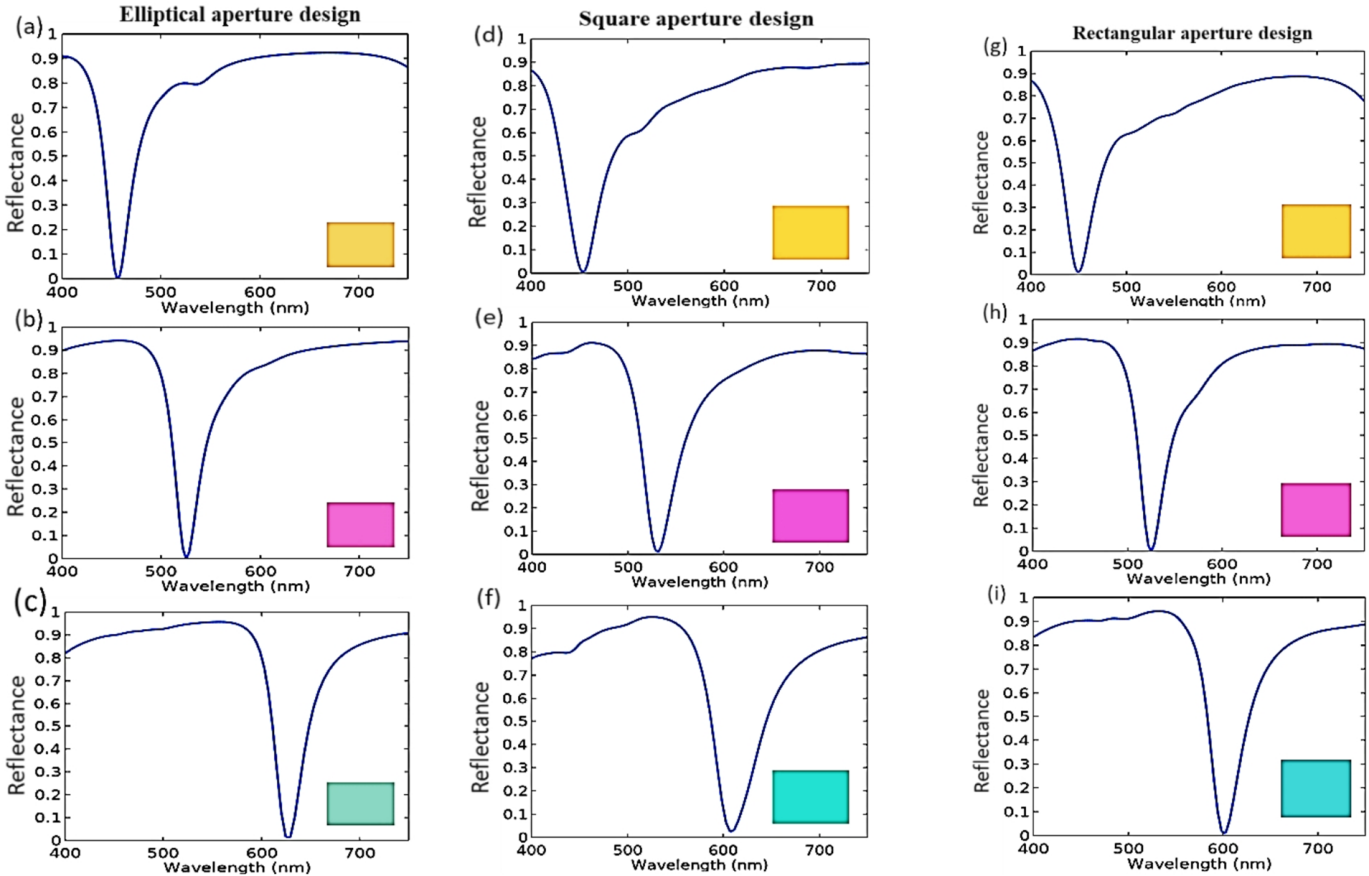
The resulted reflectance spectra for SCFs with elliptical aperture that produce (**a**) yellow, (**b**) magenta, and (**c**) cyan colors. For the square apertures, the SCFs are shown in (**d–f**) for yellow, magenta, and cyan colors, respectively. In the case of rectangular apertures the yellow, magenta, and cyan SCF are shown in (**g–i**).

The obtained reflectance spectra of the SCFs with elliptical nano-apertures for yellow, magenta, and cyan are shown in Fig. 4a–c. In this study, the design parameters are the period (P), spacer thickness (S), the gap depth (H), and the inner semi-major axis ($\:{a}_{y}$) with the values of (*P* = 250 nm, S = 100 nm, $\:{a}_{y}$ = 30 nm, H = 35 nm, $\:{w}_{1}$ = 5 nm), (*P* = 270 nm, S = 125 nm, $\:{a}_{y}$ = 60 nm, H = 32, $\:{w}_{1}$ = 10 nm), and (*P* = 300 nm, S = 160 nm, $\:{a}_{y}$ = 90 nm, H = 30 nm, $\:{w}_{1}$ = 10 nm) with the corresponding absorption resonance wavelengths of 455 nm, 525 nm, and 627 nm for yellow, magenta, and cyan, respectively. In addition, the semi- minor axis ($\:{a}_{x}$) is fixed at 20 nm for all the suggested SCFs designs.

Figure 4d–f show the reflected spectra of yellow, magenta, and cyan SCFs in the case of the square aperture. In this study, the parameters of the proposed filters have the values of period P (200, 210, 260) nm, height H (30, 25, 20) nm, spacer height S (95, 125, 150) nm, and the inner side length $\:{L}_{0}$ (165, 185, 210) nm with the corresponding absorption resonance wavelengths are 455 nm, 531 nm, and 609 nm for yellow, magenta, and cyan, respectively. Also, air gap width $\:{w}_{0}$ is fixed at 15 nm for all SCFs. Figure 4g–i show the results for yellow, magenta, and cyan SCFs obtained by the rectangular aperture metasurface design. In this study, the design parameters of the filters have the values of P (190, 235, and 300) nm, H (30, 30, and 25) nm, and the long side in the y direction $\:{L}_{y}$ (160, 185, and 210) nm, S (95, 125, and 150) nm for yellow, magenta, and cyan, respectively. Also, the inner short side is fixed at a value of 130 nm for all designs with the cavity width ($\:{w}_{3}$) remains fixed at 10 nm. The associated absorption resonance wavelengths are 450, 525, and 602 nm for yellow, magenta, and cyan, respectively.

**Fig. 5:**
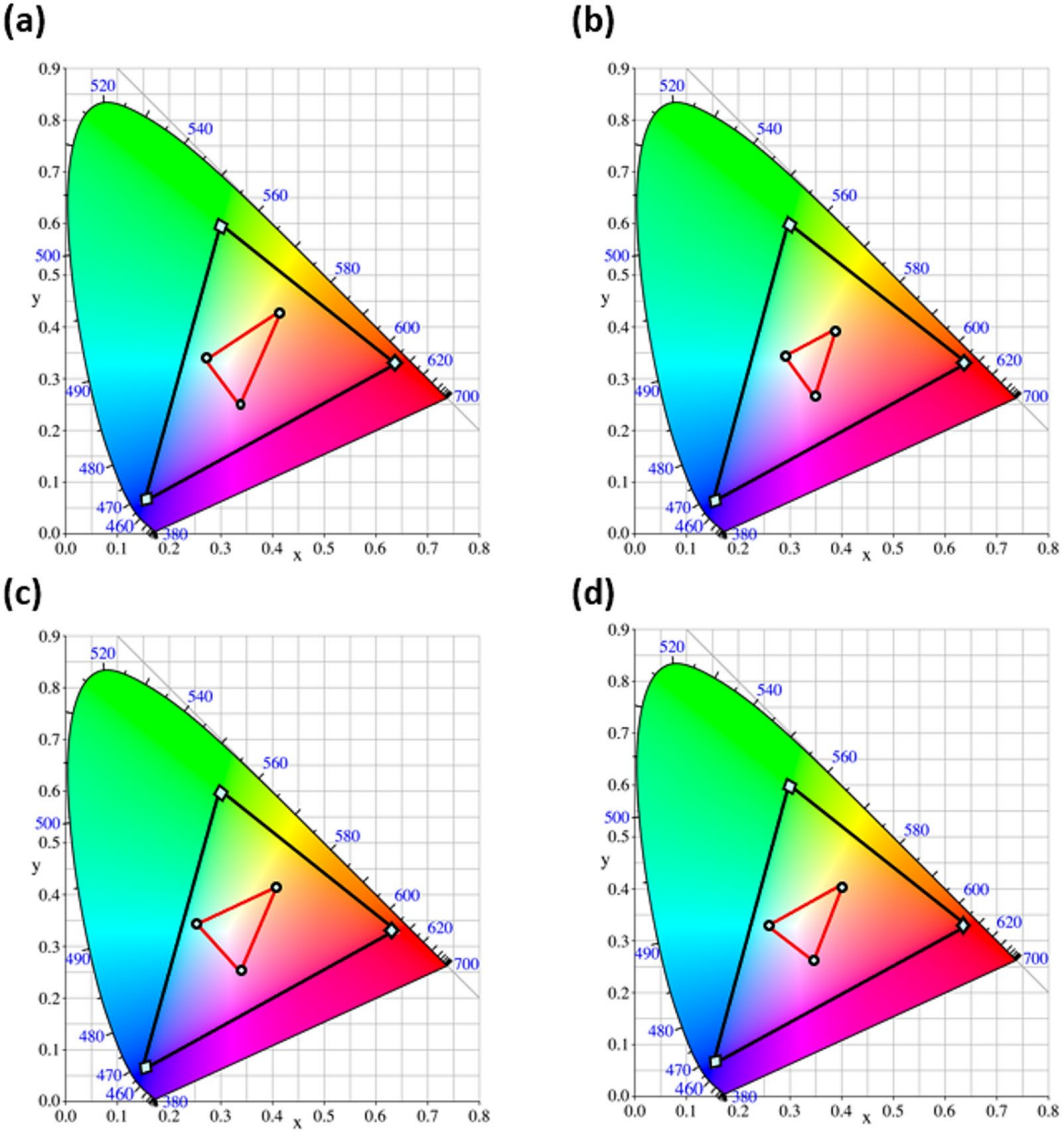
CIE 1931 chromaticity diagram comparing the simulated color gamuts of the proposed SCFs designs (red triangles) with the standard sRGB reference gamut (black triangles) for (**a**) circular, (**b**) elliptical, (**c**) square, and (**d**) rectangular apertures.

The chromatic appearance of each SCF spectrum is quantified using the CIE 1931 chromaticity diagram where the resulting coordinates for the subtractive primaries for all aperture designs are summarized in Supplementary Table S1. Figure 5 presents the chromaticity diagrams for the proposed SCFs, comparing their simulated color gamuts (red triangles) against the standard sRGB reference gamut (black triangles). Notably, the performance of subtractive plasmonic color filters is effectively characterized by the Excitation Purity ($\:{\mathrm{P}}_{\mathrm{e}}$) and Spectral Contrast Ratio (CR). The Excitation Purity is defined as the ratio of the distance from the D65 white point (.

, $\:{y}_{w}$) = (0.3127, 0.3290) to the color coordinate ($\:x$, $\:y$), relative to the distance to the spectrum locus ($\:{x}_{d}$, $\:{y}_{d}$) along the same hue line, based on the following equation^79^.

2 $$\:{P}_{e}=\sqrt{\frac{{\left(x-{x}_{w}\right)}^{2}+{\left(y-{y}_{w}\right)}^{2}}{{\left({x}_{d}-{x}_{w}\right)}^{2}+{\left({y}_{d}-{y}_{w}\right)}^{2}}}\times\:100$$

Furthermore, the sRGB coverage is determined by the area of the triangle formed by the three subtractive primaries ($\:{\mathrm{S}\mathrm{C}\mathrm{F}}_{area}$) divided by the area of the standard sRGB triangle ($\:{\mathrm{s}\mathrm{R}\mathrm{G}\mathrm{B}}_{area}$= 0.11205), and is given by^80^.

3 $$\:\mathrm{G}\mathrm{a}\mathrm{m}\mathrm{u}\mathrm{t}\:\mathrm{C}\mathrm{o}\mathrm{v}\mathrm{e}\mathrm{r}\mathrm{a}\mathrm{g}\mathrm{e}\:=\:\frac{{\mathrm{S}\mathrm{C}\mathrm{F}}_{area}}{{\mathrm{s}\mathrm{R}\mathrm{G}\mathrm{B}}_{area}}\:\times\:100$$

To evaluate the optical resonance depth and spectral purity, we define the Spectral Contrast Ratio (CR) as the ratio of the Background Reflectance ($\:{R}_{bg}$) to the Minimum Reflectance ($\:{\mathrm{R}}_{\mathrm{m}\mathrm{i}\mathrm{n}}$). This metric determines the chromatic distinction and the efficiency of the resonance-induced spectral modulation. Complementarily, the Background Reflectance Level (BRL) represents the average reflectance in the non-resonant regions of the visible spectrum, serving as a critical metric for the luminous brightness of the SCF. Our proposed structures exhibit exceptional optical performance as demonstrated in Supplementary Table S2, with near-unity absorption efficiencies exceeding 99.9% and high quality factors (Q) reaching 19.60 (Q = $\:{{\uplambda\:}}_{\mathrm{r}\mathrm{e}\mathrm{s}}$ / FWHM). These features lead to a profound suppression of reflectance minima ($\:{\mathrm{R}}_{\mathrm{m}\mathrm{i}\mathrm{n}}$ < 0.1) and narrow resonance bandwidths (FWHM) as low as 31.64 nm which it is collectively minimize spectral crosstalk. Consequently, the circular aperture yields a remarkable maximum yellow purity of 56.50%, representing a significant 41.3% improvement over the elliptical design (39.99%). Similarly, the square aperture achieves a superior cyan purity of 20.86%, demonstrating a substantial 191.7% enhancement over the elliptical structure (7.15%). These results are combined with Contrast Ratios peaking at 1268.28:1, which is validating the dual optimization of high color saturation and brightness in these SCFs. The procedure used for calculating chromaticity is obtained from Ref^78^.

**Fig. 6:**
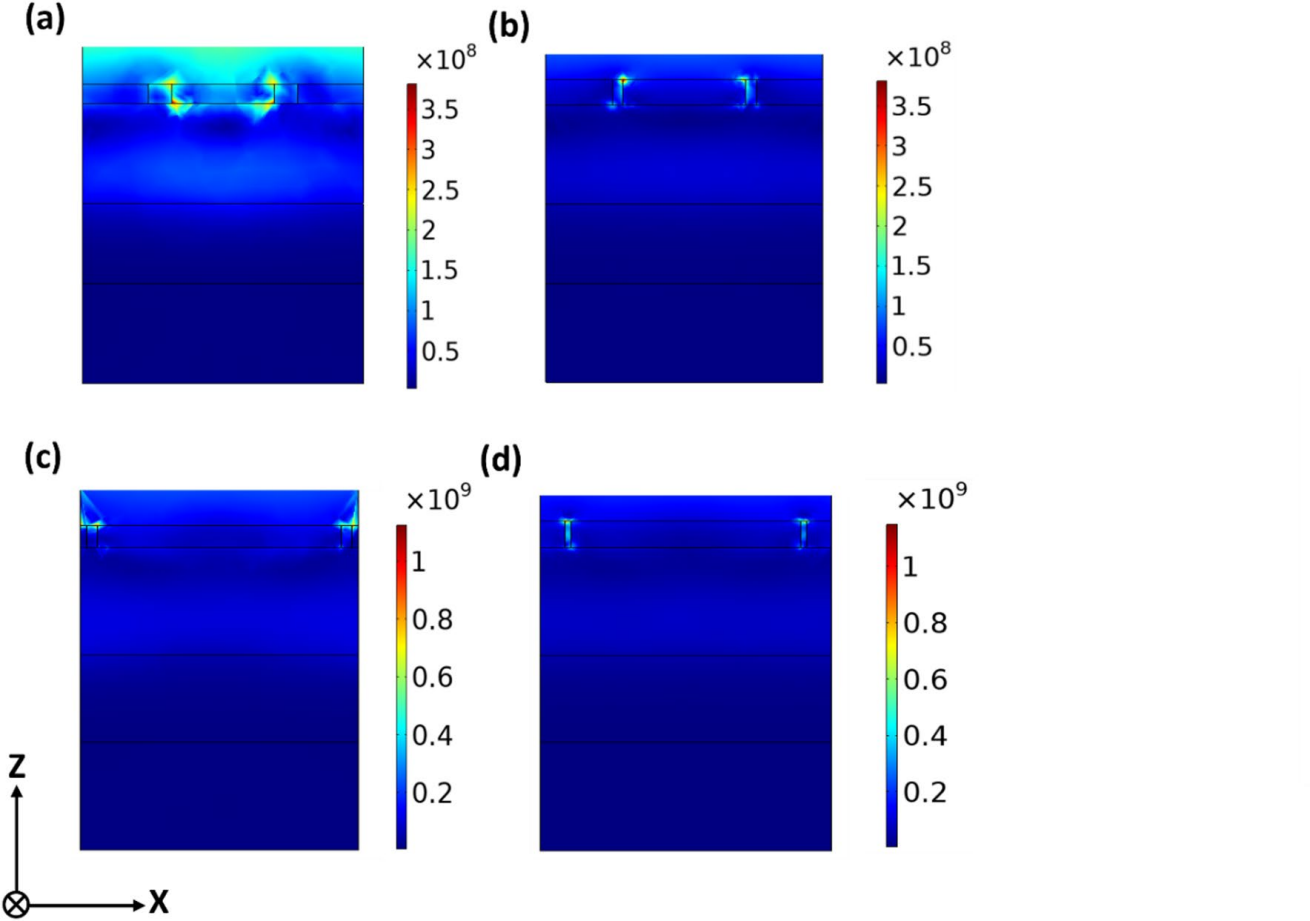
Normalized total *E*-field intensity profile, along the cross-sectional plane (*x–z* plane), (**a**) circular, (**b**) elliptical, (**c**) rectangular, and (**d**) square apertures, for the proposed filters (the magenta structure).

In order to achieve a better understanding of the CSPs and PSPs hybridization mode, the normalized total electric field intensity ($\:\mathrm{E}=\sqrt{{\left|{\mathrm{E}}_{\mathrm{x}}\right|}^{2}+{\left|{\mathrm{E}}_{\mathrm{y}}\right|}^{2}+{\left|{\mathrm{E}}_{\mathrm{z}}\right|}^{2}}\:$) is shown in Fig. 6. The strong field confinement at the aperture edges and the sharp corners confirms the excitation of CSPs mode within the annular gap, whereas the enhanced field inside the dielectric spacer reveals the coupling to PSPs mode. The E-field intensity profile is predominantly localized at the Ag-spacer layer interface and the gap, confirming the excitation of localized electric dipolar resonances.

**Fig. 7:**
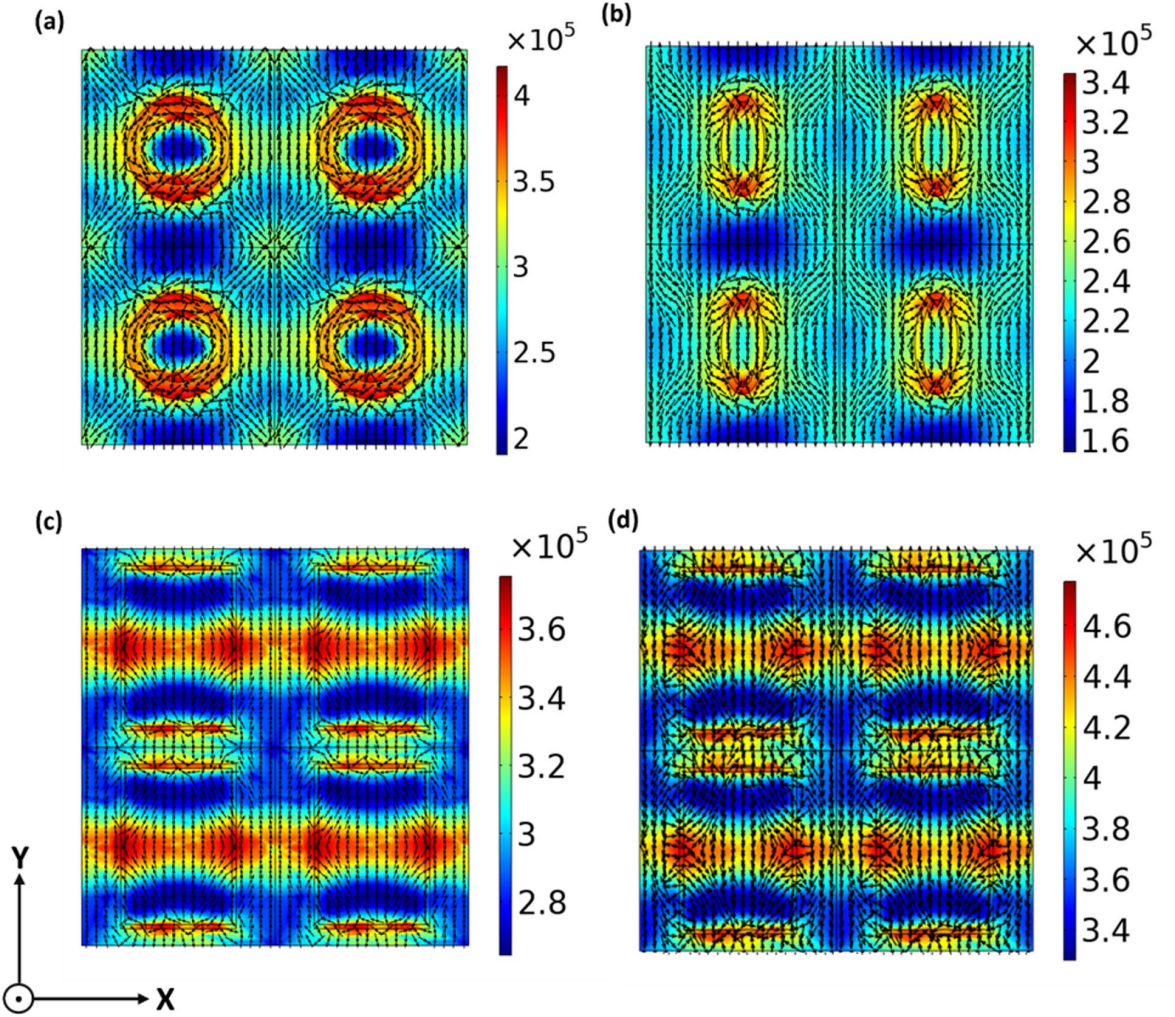
At the magenta resonance wavelength, *H*-field intensity, and displacement current ($\:{J}_{d}$), through a 2 × 2 segment of (**a**) circular, (**b**) elliptical, (**c**) rectangular, and (**d**) square apertures, showing the resonance mode. H-field distributions overlaid with the $\:{J}_{d}\:$vectors (black arrows).

Simultaneously, a significant enhancement of the H-field intensity ($\:\mathrm{H}\sqrt{{\left|{\mathrm{H}}_{\mathrm{x}}\right|}^{2}+{\left|{\mathrm{H}}_{\mathrm{y}}\right|}^{2}+{\left|{\mathrm{H}}_{\mathrm{z}}\right|}^{2}}\:$) is observed within the apertures and Ag-spacer interface which is further substantiated by the formation of displacement current $\:{\mathrm{J}}_{\mathrm{d}}\:$loops. These looping currents are represented by the vector flow in Fig. 7 and drive a strong magnetic dipolar resonance. The PSPs modes are observed to propagate largely along the diagonal directions.

The hybridization between CSPs and PSPs originates from the intersection of the diagonal PSP propagation paths with the CSP resonances. As illustrated in Fig. 7, the PSP modes propagate along the diagonal directions of the square lattice while the CSP modes are localized in the annular gaps at the sharp corners. Their spatial overlap creates coupled hybrid modes that enhance field confinement, facilitating near-perfect impedance matching, and resulting in exceptional absorption efficiencies up to 99.93%.

**Fig. 8:**
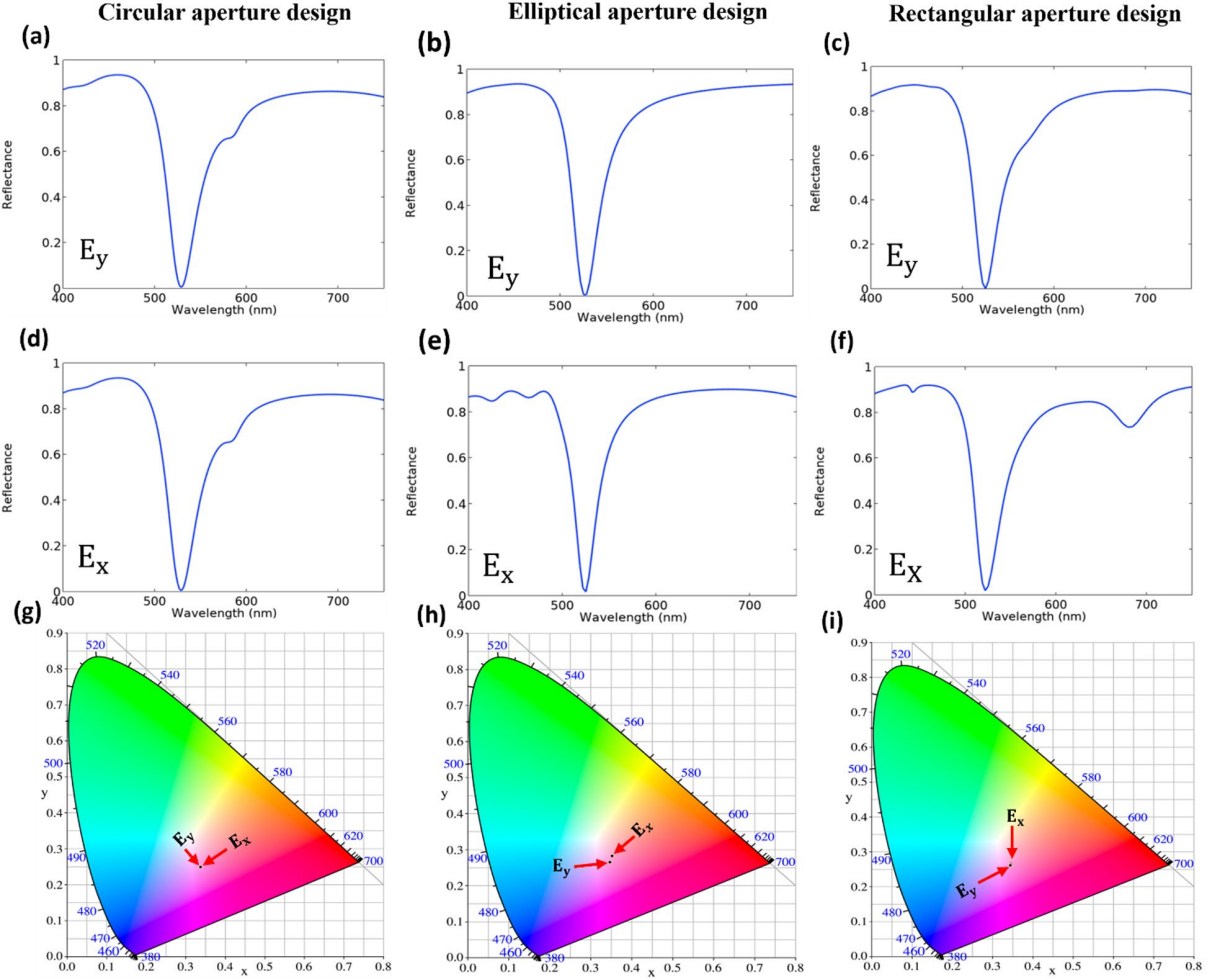
Polarization-dependent spectral response and chromaticity analysis of the proposed SCFs at the magenta resonance. (**a-c**) reflectance spectra for $\:{E}_{y}$ polarization, (**d-f**) for $\:{E}_{x}$ polarization. (**g-i**) the corresponding CIE 1931 chromaticity coordinates depending on the $\:{E}_{x}$ and $\:{E}_{y}$ polarization states.

To investigate the polarization dependence systematically, we performed a polarization-dependent analysis for both symmetric and asymmetric aperture geometries, under two orthogonal polarization states, $\:{E}_{y}$ (aligned with the major axis) and $\:{E}_{x}$ (aligned with the minor axis) as summarized in Table 2. For the circular design, the optical response remains remarkably stable where the chromaticity coordinates for both $\:{E}_{x}$ and $\:{E}_{y}$ states are nearly coincident on the CIE 1931 diagram as shown in Fig. 8g. This is maintaining a high absorption efficiency of 99.3% and a negligible purity shift.

In contrast, the asymmetric designs exhibit distinct anisotropic behaviors. For the rectangular aperture, the color coordinates show a very slight shift as indicated in Fig. 8i. This is resulting in a moderate purity variation of 1.74% while maintaining superior absorption efficiency as indicated in Table 2. The elliptical aperture shows the strongest anisotropy which it is demonstrating a significant shift between the two polarization states as presented in Fig. 8h. According to the numerical data in Table 2, this design achieves the largest shifting range in excitation purity with a significant difference of approximately 10.56%.

**Table 2:** A comparison of absorption efficiency and excitation purity for circular, elliptical, and rectangular aperture designs under $\:{E}_{x}$ and $\:{E}_{y}$ polarization conditions.

Aperture designs	Absorption efficiency (%)		$\:{P}_{e}$ (%)	
	x	y	x	y
Circular aperture	99.3	99.31	34.67	34.79
Elliptical aperture	98.3	99.3	20.09	30.65
Rectangular apertures	98.04	99.86	30.04	31.78

**Fig. 9:**
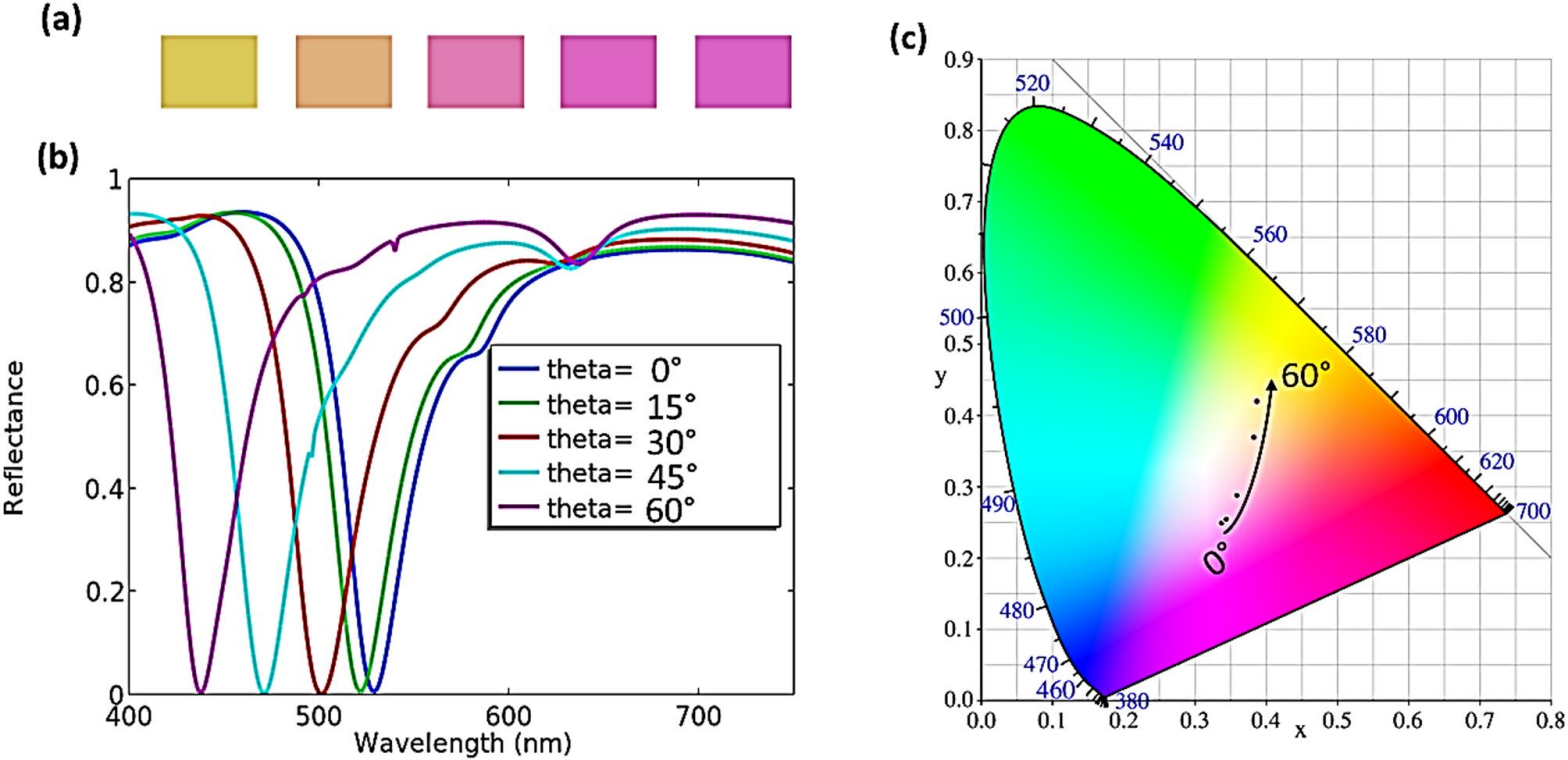
(**a**) Color images of the Circular aperture structure (magenta color design). (**b**) Simulated reflected spectra of filters. (**c**) Chromaticity coordinates in the CIE 1931 chromaticity diagram corresponding to the reflected spectra depending on the incidence angle. The incidence angle ranges from 0° to 60°.

**Fig. 10:**
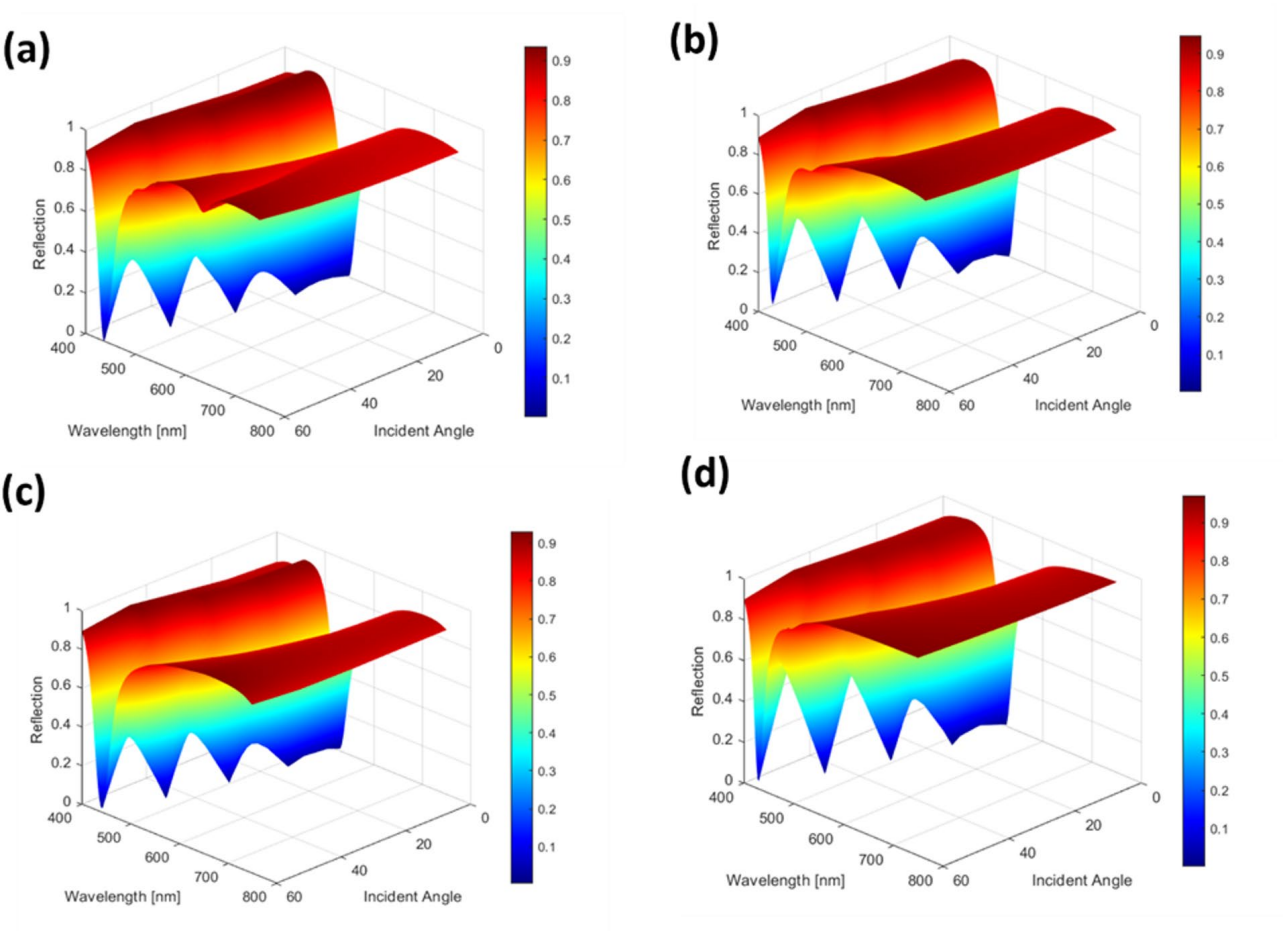
Angle-resolved reflection spectra of (**a**) circular, (**b**) elliptical, (**c**) square, and (**d**) rectangular aperture structures (magenta color design), within an angular range of 0°–60°.

The angular response of the proposed coaxial aperture structure was systematically investigated by simulating the reflectance spectra for incident angles ranging from 0° to 60° in steps of 15°, as illustrated in Fig. 9b. As the incident angle increases, the resonance wavelength exhibits a progressive blue shift as indicated in Fig. 9c. From 0° to 30°, the shift is barely perceptible, and the reflectance spectra remain nearly identical with the color appearance retaining the same magenta hue. At 30°, a slight blue shift becomes observable which slightly reduces the color purity, but the overall hue remains magenta, as shown in Fig. 9a. For larger angles (45° and 60°), the blue shift becomes more pronounced, leading to a clearly noticeable change in the perceived color. This behavior is clearly visualized in Fig. 10, which shows the evolution of the resonance position as a function of both wavelength and angle. These results confirm that the proposed filter maintains a relaxed angular tolerance up to 15°, which is sufficient for most reflective display and imaging applications where moderate viewing angles are required.

**Fig. 11:**
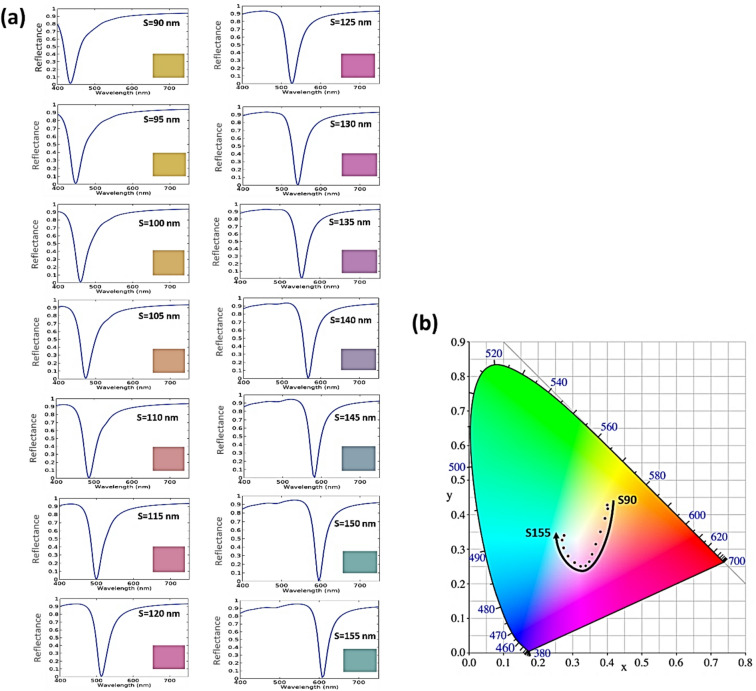
(**a**) Getting different colors by increasing the spacer thickness (S = 90–155 nm) in five steps in the case of elliptical aperture magenta SCF design. (**b**) Chromaticity coordinates in the CIE 1931 chromaticity diagram corresponding to the spectra as a function of the spacer.

We can achieve different colors from the elliptical aperture SCF design by varying the spacer thickness from 90 nm to 155 nm in the magenta SCF design. As indicated in Fig. 11, the increase in the spacer thickness results in a red shift in the reflection dip, which in turn gives the color tunability in the considered spectrum. The CIE 1931 chromaticity diagram is used to determine the colors shown in the insets of Fig. 11a.

**Fig. 12:**
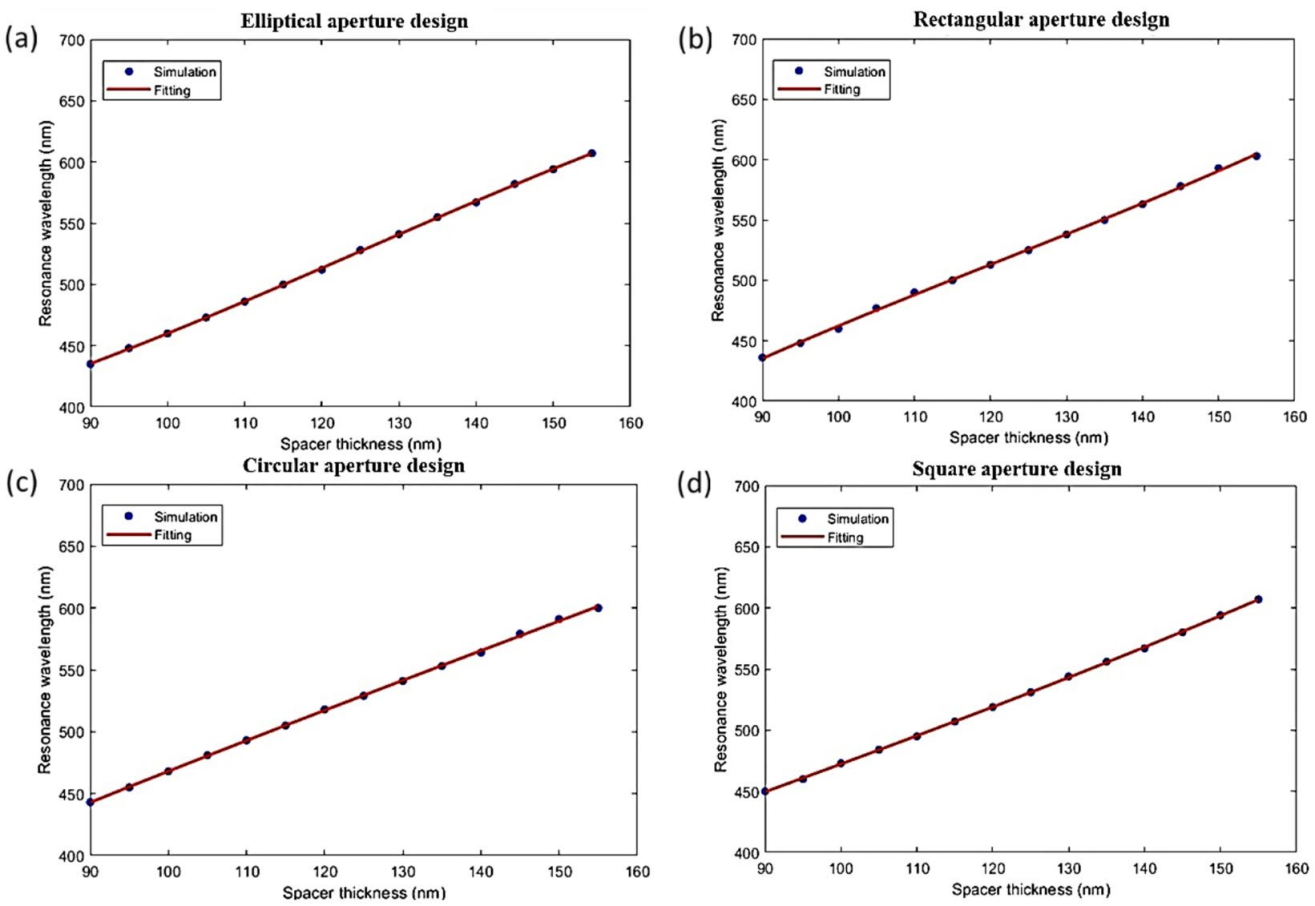
Change of resonance wavelength versus spacer thickness for the (**a**) elliptical, (**b**) rectangular, (**c**) circular, and (**d**) square apertures SCFs. The solid lines show the fitted data, while the dots show the simulated ones.

This tunability feature can be used to fit our results to a nonlinear regression model that allows a straightforward design of filters at a color of choice. Accordingly, we fitted the data from the magenta SCFs of the elliptical, circular, square, and rectangular apertures to obtain a general equation that relates the obtained resonance wavelength with changing the spacer thickness (S) of the suggested designs. We found that the resonance wavelength dependence on the spacer thickness of the proposed SCFs can be fitted to a cubic polynomial. The cubic fit gives the minimal number of coefficients required to achieve an acceptable level of accuracy as follows.

4 $$\:{\uplambda\:}={a}_{1}{S}^{3}+{b}_{1}{S}^{2}+{c}_{1}S+{d}_{1}$$

where λ is the SCF resonance wavelength, and S is the spacer thickness for the SCF structures. The symbols $\:{a}_{1}$, $\:{b}_{1}$, $\:{c}_{1}$, and $\:{d}_{1}$ are referring to the fitting coefficients with values are indicated in Table 3. The fitting of the resonant wavelengths in Fig. 12 with Eq. (4) has an average root mean square error (RMSE) of 0.7156%, 1.0344%, 0.65915%, and 1.0344% for SCFs based on elliptical, circular, square, and rectangular apertures, respectively.

**Table 3:** Fitting coefficients values of the Eq. (4).

Aperture name	$\:{a}_{1}$	$\:{b}_{1}$	$\:{c}_{1}$	$\:{d}_{1}$
Elliptical	− 0.0001	0.0386	–0.0848	385.3297
Circular	0.0000	-0.0068	3.3970	180.8931
Square	0.000	-0.0054	2.6790	235.2258
Rectangular	0.001	–0.0360	6.8487	38.1319

**Fig. 13:**
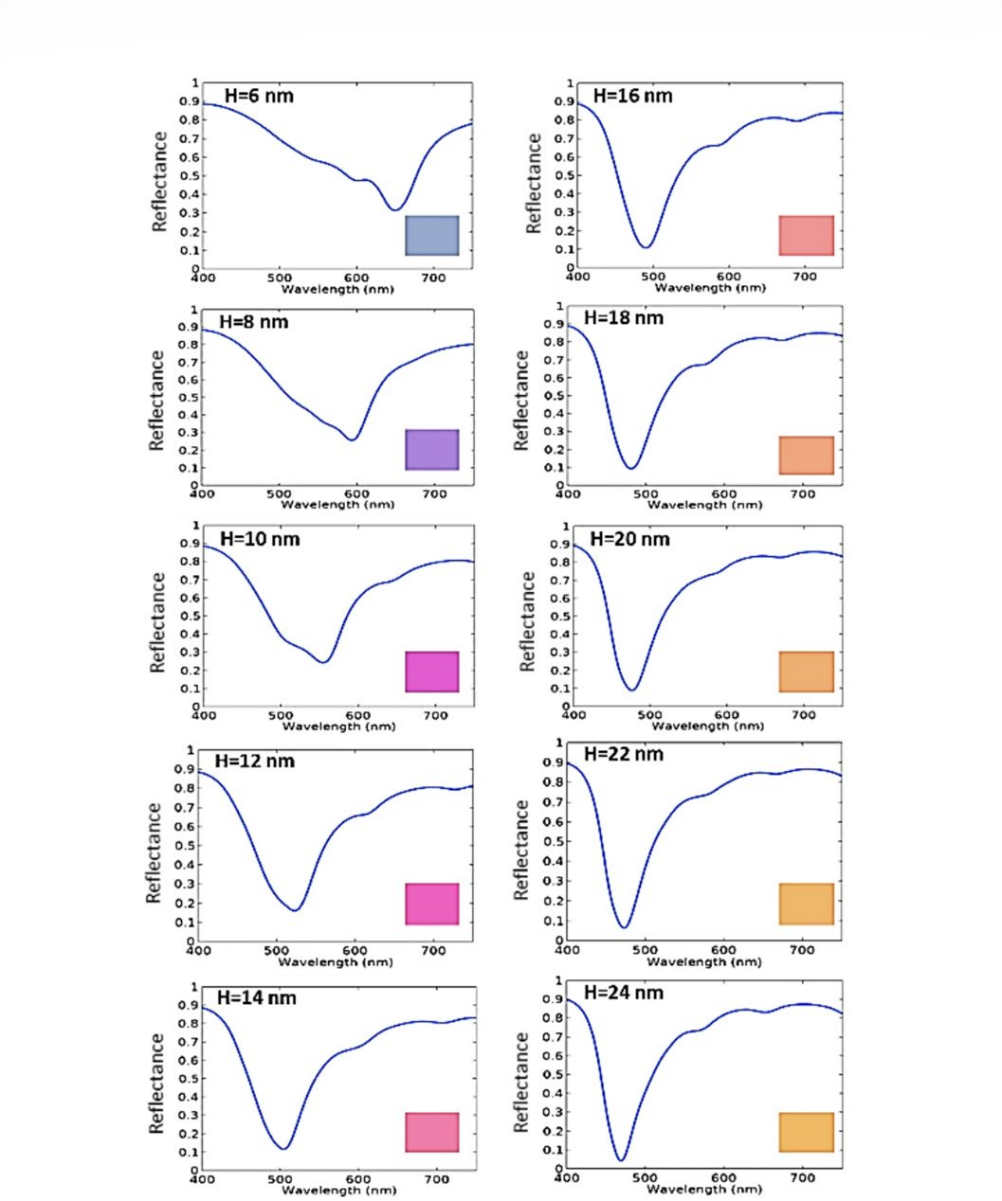
The change of the resulting colors with the cavity depth of the rectangular aperture design.

Finally, for the cavity depth (H) dependence, we found that as the cavity depth increases from 6 nm to 24 nm in two steps, the reflection dip exhibits a blue shift, confirming the color tunability as shown in Fig. 13. Here, the investigation is done for the proposed SCF designs, with design parameters indicated in Table 4. A general equation between the resultant resonance wavelength and the cavity depth (H) was obtained by fitting the simulated resonance wavelengths to their corresponding cavity depths for rectangular, circular, and square apertures SCFs designs. We found that the resonance wavelength dependence on cavity depth of the AAAs can be fitted to a quadratic exponential fitting as follows.

5 $$\:{{\uplambda\:}={a}_{2}e}^{{b}_{2}H}+{{c}_{2}e}^{{d}_{2}H}$$

**Table 4:** Design parameters of the apertures structures.

Metasurface design	Designs parameters	Value (nm)
Rectangular	*P*	200
	S	100
	$\:{L}_{x}$	130
	$\:{L}_{y}$	140
	$\:{w}_{3}$	10
Circular	P	175
	S	110
	R	65
	r	50
Square	P	220
	S	110
	$\:{L}_{0}$	165
	$\:{w}_{2}$	10

A quadratic exponential fitting supports the minimal set of coefficients for a given acceptable accuracy when the rate of change decreases exponentially. From Eq. (5), λ is the resonance wavelength and H is the cavity depth, the symbols $\:{a}_{2}$,$\:\:{b}_{2}$,$\:\:{c}_{2}\:$and$\:\:{d}_{2}$ are referring to the fitting coefficients with values are indicated in Supplementary Table S3.

The resulted fitted data compared to the simulated ones are shown in Supplementary Fig. S2. for (a) rectangular, (b) square, (c) circular apertures SCFs. From these figures, we can notice that the simulated and fitted data match each other with RMSE values of 0.8785%, 3.0607% and 3.3641% for rectangular, square, and circular apertures SCFs designs, respectively.

The absorption resonance wavelength is related to S and H of our plasmonic SCFs by the following equation (Fabry–Perot resonance and the cavity depth dependence)^81^.

6 $$\:{{\uplambda\:}}_{\mathrm{r}\mathrm{e}\mathrm{s}}=\frac{2\mathrm{L}{\mathrm{n}}_{\mathrm{g}\mathrm{s}\mathrm{p}}}{\mathrm{m}}$$

Where $\:{{\uplambda\:}}_{\mathrm{r}\mathrm{e}\mathrm{s}}$ is the resonance wavelength modulated by resonant mode numbers m. The parameter $\:\mathrm{L}$ is representing the cavity length, $\:{\mathrm{n}}_{\mathrm{g}\mathrm{s}\mathrm{p}}$ is the gap plasmon effective index. Increasing $\:\mathrm{S}$ directly extends the optical path length, which leads to a conventional red shift shown in Fig. 11. This behavior is represented by a cubic polynomial regression model.

Conversely, by increasing the cavity depth H, induces a blue-shift, which can be analytically explained by the cavity depth dependence of the effective index^82^.

7 $$\:\:\:{\mathrm{n}}_{\mathrm{g}\mathrm{s}\mathrm{p}}=\mathrm{n}-{\upalpha\:}{\Delta\:}\mathrm{n}\:$$

Where n is the material refractive index, $\:{\upalpha\:}$ is the gap depth dependence factor, and $\:{\Delta\:}\mathrm{n}\:\mathrm{i}\mathrm{s}\:$the effective refractive index change. As H increase leads to a higher depth-dependence factor ($\:{\upalpha\:}$), resulting in the reduction of $\:{\mathrm{n}}_{\mathrm{g}\mathrm{s}\mathrm{p}}$. The resulting effective refractive index is related to the resonance shifted wavelength by the following expression^82^.

8 $$\:\frac{{\mathrm{n}}_{\mathrm{g}\mathrm{s}\mathrm{p}}}{{\Delta\:}\mathrm{n}}=\frac{{{\uplambda\:}}_{\mathrm{r}\mathrm{e}\mathrm{s}}}{{\Delta\:}{{\uplambda\:}}_{\mathrm{r}\mathrm{e}\mathrm{s}}}$$

The reduction in effective refractive index with depth is providing a negative wavelength shift corresponding to a blue shift. This behavior is represented by a quadratic exponential model. Moreover, as the cavity depth increases, the electromagnetic field strongly confined within the annular gap, enhancing the light matter interaction and leading to a significant increase in absorption efficiency, as shown in Fig. 13. Furthermore, the spectral bandwidth (FWHM) of this resonance is calculated by^81^

9 $${\rm FWHM} \:\approx\:\frac{\delta\:{{{\uplambda\:}}_{\mathrm{r}\mathrm{e}\mathrm{s}}}^{2}}{{\mathrm{n}}_{\mathrm{g}\mathrm{s}\mathrm{p}}}$$

where $\:\delta\:$ is the effective absorption coefficient. As illustrated in Fig. 13, increasing the cavity depth $\:H$ induces a blue shift. This shift is accompanied by a narrowing of the resonance peak, which provides an improved quality factor ($\:Q={\lambda\:}_{\mathrm{r}\mathrm{e}\mathrm{s}}/\mathrm{F}\mathrm{W}\mathrm{H}\mathrm{M}$) and enhanced light matter interaction.

## Conclusion

In summary, we have proposed and numerically demonstrated various designs of the plasmonic metasurfaces for the generation of SCFs. We have investigated the effectiveness of silver-based coaxial aperture arrays as SCFs operating in the reflection mode. The resulted colors achieved using plasmonic resonance has intriguing features such as exceedingly high saturation and brightness. In addition, we showed that the demonstrated approach can be easily applied to various shapes of plasmonic nanoapertures, such as circular, elliptical, rectangular, and square nanoapertures. The proposed devices exhibit a polarization-insensitive operation for symmetric apertures and a polarization-sensitive response for asymmetric apertures, and a relaxed angular tolerance. Color tunability over the entire visible spectrum can be improved through controlling the geometry of the plasmonic nanoapertures. As a result, we used an analytical expression to generalize the concept of obtaining the SCFs over the visible spectrum in a straightforward way. We expect that the approach can facilitate the design of SCFs opening up opportunities for practical applications such as color printing, sensing, cryptography, imaging, optical data storage, further optical devices and high-resolution chromatic displays.

## Supplementary Information

Below is the link to the electronic supplementary material.

Supplementary Material 1

## Data Availability

Requests for data and materials should be addressed to corresponding author e-mail : waliedsabra@science.bsu.edu.eg.
