# Supplementary material for: Subtractive color filters based coaxial metasurface structures with high saturation and brightness
Source: Sci Rep. 2026 May 13;16:15037. doi: 10.1038/s41598-026-51341-0 (PMC13172545; doi:10.1038/s41598-026-51341-0)
Supplement: Supplementary file 1 — Supplementary Material 1 [file 41598_2026_51341_MOESM1_ESM.pdf]

Supplementary Information

Subtractive Color Filters Based Coaxial Metasurface Structures with High Saturation and Brightness

Abdelnaser Ali [1], Hassan Sayed[1], Mohamed Mobarak [2], Arafa H Aly[1], and Walied Sabra [1, $]

[1] *TH-PPM Group, Physics Department, Faculty of Science, Beni-Suef University, Egypt*
[2] *Physics Department, Faculty of Science, Beni-Suef University, Egypt*

[$] Corresponding Author E-mail: waliedsabra@science.bsu.edu.eg

This document contains the following supporting information:

1. Numerical Analysis of the displacement current and impedance coefficient, to verify the resonance conditions.

2. Extended Performance Metrics, including comprehensive data tables for CIE 1931 coordinates (x, y), color purity (P_e), Q-factors, and absorption efficiencies across various aperture geometries.

3. Polynomial Regression and Fitting Parameters corresponding to the numerical analysis of the resonance wavelength dependence on cavity depth (H)

1. Numerical Analysis of the displacement current and impedance coefficient, to verify the resonance conditions.

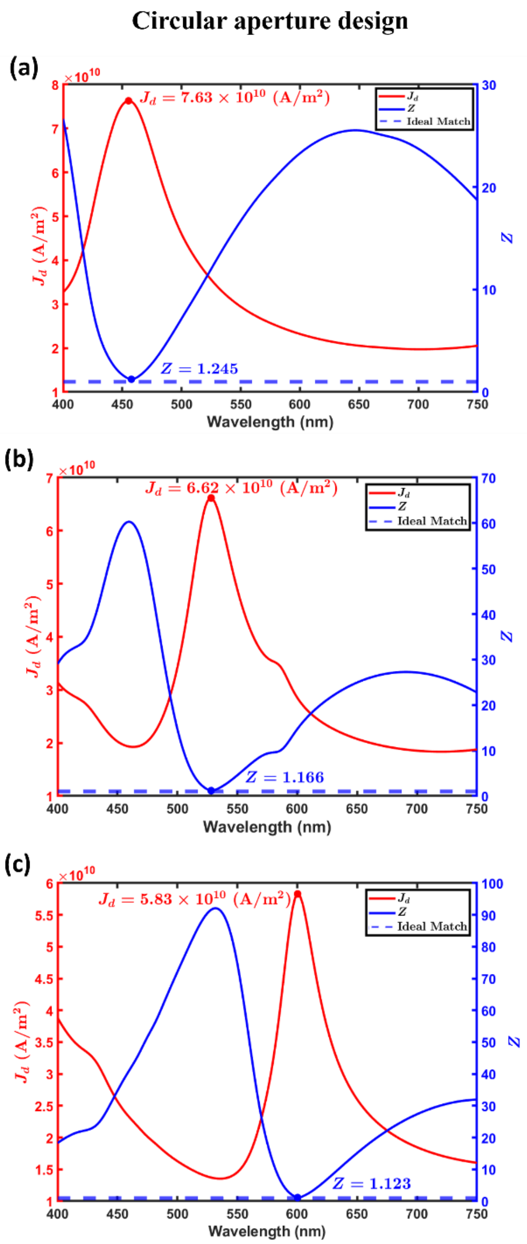

Fig. S1. The calculated impedance coefficient Z (blue curves) and the displacement current density J_d (red curves) spectra for the circular aperture SCFs, (a) yellow, (b) magenta, and (c) cyan designs.

2. Extended Performance Metrics, including comprehensive data tables for CIE 1931 coordinates (x, y), color purity (P_e), Q-factors, and absorption efficiencies across various aperture geometries.

Table S1. CIE 1931 coordinates, color purity, and sRGB gamut coverage for the proposed coaxial aperture designs.

Aperture designs	Color	CIE_X	CIE_Y	P_e (%)	sRGB Coverage (%)
Circular aperture	Yellow	0.415	0.4286	56.50	8.16
	Magenta	0.3367	0.2500	34.69	
	Cyan	0.2717	0.3352	14.52	
Elliptical aperture	Yellow	0.3884	0.3962	39.99	4.73
	Magenta	0.3462	0.2649	30.65	
	Cyan	0.2909	0.3442	7.15	
Square apertures	Yellow	0.4089	0.414	50.71	9.07
	Magenta	0.3371	0.2503	34.64	
	Cyan	0.2531	0.3419	20.86	
Rectangular apertures	Yellow	0.4031	0.4094	47.80	7.37
	Magenta	0.3441	0.2610	31.78	
	Cyan	0.2590	0.3267	19.74	

Table S2. Detailed performance metrics of the proposed coaxial SCF designs across different aperture geometries for the subtractive primary colors.

Aperture designs	Color	R_{min} (%)	Absorption Efficiency (%)	FWHM	Q-factor	BRL (%)	CR
Circular aperture	Yellow	1.19	98.81	55.33	8.27	83.87	70.47:1
	Magenta	0.59	99.41	41.12	12.84	88.19	149.47:1
	Cyan	0.34	99.66	33.90	17.71	88.98	261.7:1
Elliptical aperture	Yellow	0.24	99.76	31.64	14.39	89.25	371.87:1
	Magenta	0.68	99.32	35.62	14.74	90.73	133.42:1
	Cyan	1.45	98.55	32.09	19.60	90.35	62.31:1
Square apertures	Yellow	0.55	99.45	47.98	9.44	86.37	157.03:1
	Magenta	1.36	98.64	43.17	12.31	86.98	63.95:1
	Cyan	2.5	97.50	52.36	11.59	85.95	34.38:1
Rectangular apertures	Yellow	1.32	98.68	42.71	10.55	83.98	63.62:1
	Magenta	0.07	99.93	35.62	14.74	88.78	1268.28:1
	Cyan	1.09	98.91	42.39	14.21	89.17	81.8:1

3. Polynomial Regression and Fitting Parameters corresponding to the numerical analysis of the resonance wavelength dependence on cavity depth (H)

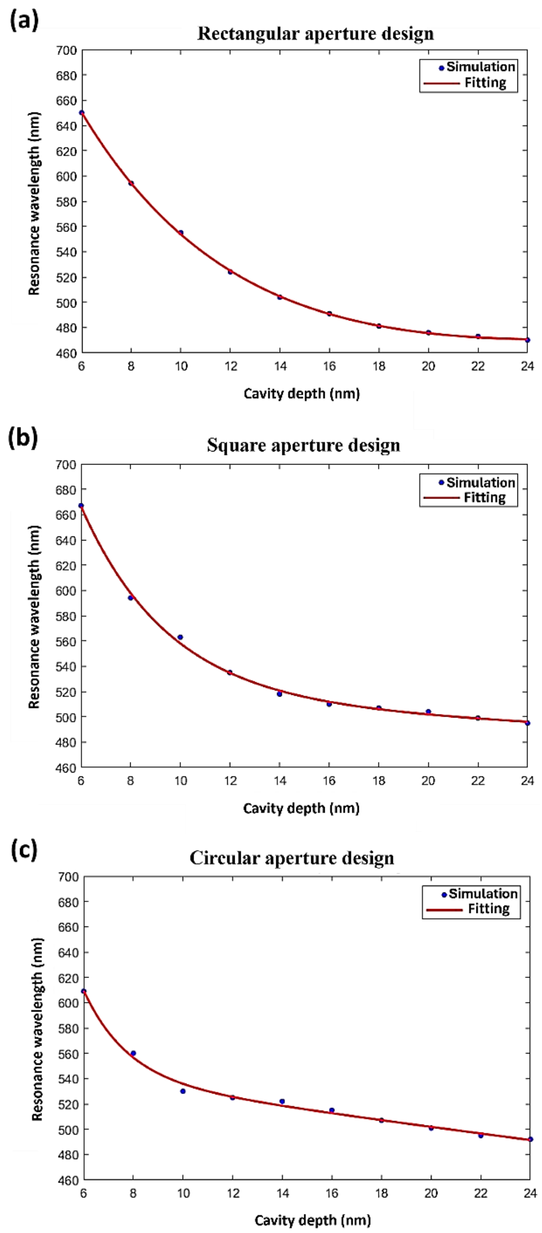

Fig. S2. Change of resonance wavelength versus the cavity depth of the AAAs of (a) rectangular, (b) square, and (c) circular SCFs designs, respectively. The solid lines show the fitted data while the dots represent simulated data.

Table S3. Fitting coefficients values of the equation (5)

Aperture Name	a_2	b_2	c_2	d_2
Rectangular	565.8456	-0.1522	412.6288	0.0042
Square	845.1249	-0.2845	519.0092	-0.002
Circular	2.1279×10^3	-0.5702	557.2273	-0.0052
